# Understanding Cultivar-Specificity and Soil Determinants of the *Cannabis* Microbiome

**DOI:** 10.1371/journal.pone.0099641

**Published:** 2014-06-16

**Authors:** Max E. Winston, Jarrad Hampton-Marcell, Iratxe Zarraonaindia, Sarah M. Owens, Corrie S. Moreau, Jack A. Gilbert, Josh Hartsel, Suzanne J. Kennedy, S. M. Gibbons

**Affiliations:** 1 The Field Museum, Department of Science and Education, Chicago, Illinois, United States of America; 2 Committee on Evolutionary Biology, University of Chicago, Chicago, Illinois, United States of America; 3 Argonne National Laboratory, Institute for Genomic and Systems Biology, Lemont, Illinois, United States of America; 4 Department of Ecology and Evolution, University of Chicago, Chicago, Illinois, United States of America; 5 Basque Country Government, Bilbao, Spain; 6 Computation Institute, University of Chicago, Chicago, Illinois, United States of America; 7 Cannavest, San Diego, California, United States of America; 8 MO BIO Laboratories, Carlsbad, California, United States of America; 9 Graduate Program in Biophysical Sciences, University of Chicago, Chicago, Chicago, Illinois, United States of America; Graz University of Technology (TU Graz), Austria

## Abstract

Understanding microbial partnerships with the medicinally and economically important crop *Cannabis* has the potential to affect agricultural practice by improving plant fitness and production yield. Furthermore, *Cannabis* presents an interesting model to explore plant-microbiome interactions as it produces numerous secondary metabolic compounds. Here we present the first description of the endorhiza-, rhizosphere-, and bulk soil-associated microbiome of five distinct *Cannabis* cultivars. Bacterial communities of the endorhiza showed significant cultivar-specificity. When controlling cultivar and soil type the microbial community structure was significantly different between plant cultivars, soil types, and between the endorhiza, rhizosphere and soil. The influence of soil type, plant cultivar and sample type differentiation on the microbial community structure provides support for a previously published two-tier selection model, whereby community *composition* across sample types is determined mainly by soil type, while community *structure* within endorhiza samples is determined mainly by host cultivar.

## Introduction

Soil microbes play a major role in plant ecology by providing a variety of benefits such as nitrogen fixation, production of growth stimulants, improved water retention, and suppression of root diseases [1]–[4]. These vital microbial processes occur predominantly within the rhizosphere and rhizoplane, and are heavily influenced by fungal saprotrophs and plant-mutualists such as endomycorrhizal and ectomycorrhizal fungi [5], [6]. Despite the economic and medicinal importance of *Cannabis spp.*, little is known about its soil-based microbial associations [7], [8].

Microbial composition in soil depends on complex interactions between the soil type, root zone location, and plant species [9]–[11]. Rhizosphere microbiota are highly dynamic [12], and the composition of bacterial communities can fluctuate in response to seasonal and diel temperature changes [13], water content [14], pH [15], CO_2_ concentration, and O_2_ levels [16]. Although evidence has been found for significant effects of plant cultivar on rhizosphere communities [17]–[19] and endomycorrhizal fungal communities [20], some work suggests that these effects are minimal compared to edaphic factors (particularly pH) or plant growth stage [21], [22].

Rhizosphere bacteria not only colonize the rhizosphere and/or the rhizoplane soil, but can also colonize plant tissues. Bacteria that have colonized root tissue—more specifically known as the endorhiza [23]—have been reported to support plant growth and suppress plant diseases by providing phytohormones, low molecular weight compounds or enzymes involved in regulating growth and metabolism [24]–[26]. In addition, endorhiza bacteria assist their host plants in tolerating the phytotoxic effects of environmental toxicants [27], [28]. Endorhiza communities tend to be more plant-specific, and are often shaped by the compounds or proteins produced by their host [29]. Both endophytes and epiphytes may also play a role in localized ‘flavor’ or *terroir* for crop plants, as has been shown recently for wines [30]–[32].

A growing body of work has united the colonization of both the rhizosphere and plant tissues under the two-tier selection model, where soil type defines the composition of rhizosphere and root-inhabiting bacterial communities [33]–[35]. Under this model, edaphic factors determine the structure of the local soil microbiota, which become the source for the first bacterial community shift into the nutrient rich environment of the rhizosphere. Following this first shift, migration from the rhizosphere into the plant tissues is based on plant genotype-dependent selection of the endorhiza environment [33]. Along with the prediction that rhizosphere and endorhiza microbiota should be soil-derived, the two-tier selection model predicts several broad changes in phylum-level taxon abundance associated with the shifting microbiota, such as dramatic reduction in *Acidobacteria* within the endosphere.

This study aims to characterize bacterial diversity in the root and soil systems of five strains of *Cannabis* in order to explore how soil microbiota and plant strain affect the endorhiza microbial community of this commercially important crop. We hypothesize that different cultivars maintain significantly different microbial communities, and that these differences diminish from endorhiza to rhizosphere to bulk soil.

## Materials and Methods

### Experiments

The data for this paper were collected in two experiments: First, an experiment to identify variation in the microbial communities, and second, an experiment designed to understand the nature and strength of cultivar-specificity. The first experiment was composed of bulk soil, rhizosphere, and endorhiza samples taken from nine plants of the three different *Cannabis* spp. tested strains—Burmese, BooKoo Kush, and Sour Diesel. Soil physicochemical data was taken for all bulk soil samples in the first experiment, however there was minimal edaphic variation. The second experiment sought to understand the effect of strain with more significant edaphic variation, and was accomplished using two different strains—White Widow and Maui Wowie—and two different soil types. Four plants of the two strains were grown in the same soil, and then two plants of White Widow were grown in a completely distinct soil type. Triplicate samples were taken from each plant for both the rhizosphere and endorhiza, as well as for each of the two soil types.

### Cultivars

Different cultivars were used for each one of the experiments. For the first experiment, we used Sour Diesel, Bookoo Kush, and Burmese cultivars. Sour Diesel is a cultivar of *Cannabis sativa*, associated with a high tetrahydrocannabinol (THC) to cannabidiol (CBD) ratio. Bookoo Kush is a sativa-dominant hybrid cultivar of *Cannabis sativa* and *Cannabis indica*, associated with a moderately high THC to CBD ratio. Burmese is a balanced hybrid cultivar of both *Cannabis sativa* and *Cannabis indica*, associated with a moderate THC to CBD ratio. For the second experiment, we used Maui Wowie and White Widow cultivars. Maui Wowie is a cultivar of *Cannabis sativa*, associated with a high THC to CBD ratio. White Widow is a balanced hybrid cultivar of both *Cannabis sativa* and *Cannabis indica*, known to have a more moderate THC to CBD ratio.

### Sample Collection

Endorhiza, rhizosphere soil, and bulk soil samples for the first experiment were taken from 9 organically-grown *Cannabis* plants of three different strains (Burmese, Bookoo Kush, Sour Diesel) in Vista, California, in November, 2011, for a total of 27 samples. Therefore, the triplicate DNA extracts were acquired for endorhiza, rhizosphere and bulk-soil for each of the 3 *Cannabis spp.* strains, resulting in a single endorhiza, rhizosphere, and bulk soil sample for each plant. The plants were grown in locally composted soil. Eight weeks following the harvesting of the *Cannabis* flowering bud and foliage from each plant, a 50 g bulk soil sample was taken 10 cm from the stem of each of the nine plants at a depth of 20 cm, as well as a larger sample of soil for testing edaphic factors (**Table 1**). The bulk soil sample was immediately capped and transported to a 4°C refrigerator. In addition, endorhiza samples were taken from the root ball of each of the six plants. The soil that remained adhered to the roots after removal from the ground was used to produce the rhizosphere soil samples. The rhizosphere soil was removed from the roots by shaking the root into a whirlpak bag. All samples were immediately transferred to storage at 4°C for shipping back to the laboratory for processing (approximately 4 hours). All root samples were rinsed with alcohol and sterile water before the extraction. DNA was isolated from 0.25 g of soil or root per extraction using standard protocol for PowerSoil DNA Isolation Kit (MO BIO, USA), with the modification of heating the extraction at 65°C for 10 minutes prior to the initial vortex step. The soil physicochemical data was generated by Fruit Growers Laboratory (Santa Paula, CA), including total carbon and nitrogen concentrations, pH, salinity, and water content for all samples.

**Table 1:** Soil Physicochemical Data.

Soil ID	Physical Composition	pH	Salinity	Total N	Total Organic C	Water Content
MB.1.B	64.6 sand, 17.6 silt, 17.8 clay	6.94	7.15	1.41	5.00	0.164
MB.1.SD	66.0 sand, 16.3 silt, 17.7 clay	6.80	7.10	1.51	4.32	0.178
MB.1.BK	63.1 sand, 17.7 silt, 19.2 clay	6.82	7.44	1.30	3.31	0.101
MB.2	62.0 sand, 17.3 silt, 20.7 clay	6.63	5.12	0.26	3.02	0.113
OC.2	64.0 sand, 16.0 silt, 20.0 clay	6.77	1.73	0.53	20.0	0.371

Physical composition and tested edaphic factors for five soil types from both experiments. Abbreviations for Soil ID are: MB indicates Mo-Bio soil, OC indicates Orange County soil, number indicates experiment (1 =  first experiment, 2 =  second experiment), and final letter abbreviations detail the associated cultivar with the bulk soil. B =  Burmese, SD =  Sour Diesel, BK =  Bookoo Kush.

Endorhiza, rhizosphere, and bulk soil samples for the second experiment were taken from 6 organically-grown *Cannabis* plants of two different strains (White Widow and Maui Wowie) from two locations in August, 2012: Vista and Orange County, California. Triplicate samples were taken from each of the six plants (18 samples) and surrounding rhizosphere (18 samples), as well as from each of the two bulk soils used in the different locations (6 samples), totaling 42 samples. In contrast to the first experiment, all samples were taken two weeks prior to harvest. Additionally, triplicate samples from the second experiment were taken from different roots on the same plant (pseudoreplicates). Cannabinoid data was taken from the buds of three White Widow plants and one Mauie Wowie plant (**Table S1**). All cannabinoid data was processed at Delta-9-Technologies, LLC (Santa Ana, California). Otherwise, sampling procedure matched the first experiment.

### Illumina sequencing of the V4 region of the 16S rRNA gene

We utilized Illumina 16S rRNA sequencing to analyze samples of the endorhiza, the rhizosphere, and the bulk soil of three different strains of *Cannabis* in the first study (27 samples), and two different strains of *Cannabis* in the second study (42 samples), for a total of 69 samples. The V4 region of the 16S rRNA gene was amplified and sequenced using the primers specified in Caporaso et al. (2012) following the Earth Microbiome Project's standard pipeline (http://www.earthmicrobiome.org/emp-standard-protocols/) [36]. The 291 bp length V4 region amplification was performed using the 515F primer and the 806R Golay–barcoded reverse primers (for a full list of these primers visit http://www.earthmicrobiome.org/emp-standard-protocols/). Each 25 µL PCR reaction contained 12 µL of MO BIO PCR Water (Certified DNA-Free), 10 µL of 5 Prime HotMasterMix (1x), 1 µL of Forward Primer (5 µM concentration, 200 pM final), 1 µL Golay Barcode Tagged Reverse Primer (5 µM concentration, 200 pM final), and 1 µL of template DNA. The conditions for PCR are as follows: 94°C for 3 minutes to denature the DNA, with 35 cycles at 94°C for 45 s, 50°C for 60 s, and 72°C for 90 s, with a final extension of 10 min at 72°C to ensure complete amplification. PCR was completed in triplicate and products were pooled. Each pool was then quantified using Invitrogen's PicoGreen and a plate reader. Once quantified, different volumes of each of the products were pooled into a single tube so an equal amount (ng) of DNA was in the pool, and cleaned using the UltraClean PCR Clean-Up Kit (MO BIO). After quantification, the molarity of the pool is determined and diluted down to 2 nM, denatured, and then diluted to a final concentration of 6.1 pM with a 30% PhiX spike for sequencing on the Illumina MiSeq. A 151 bp×12 bp×151 bp MiSeq run was performed using the custom sequencing primers and procedures described in the supplementary methods in Caporaso et al. (2012). All raw sequence data is available publicly [37].

### Bioinformatic analysis of the 16S rRNA V4 sequence data

All sequence analysis was done using QIIME 1.7.0 [38]. QIIME defaults were used for quality filtering of raw Illumina data. In the second study, both closed and open reference OTU-picking methods were employed. In the first study, OTUs were picked against the Greengenes [39] database pre-clustered at 97% identity, and sequences that did not hit the reference collection were clustered *de novo* (i.e. open reference). Representative sequences were aligned to the Greengenes core set with PyNAST [38]. All sequences that failed to align were discarded. A phylogenetic tree was built from the alignment using FastTree [40], and taxonomy was assigned to each sequence using the RDP classifier [41] retrained on Greengenes. Samples for the first experiment were rarified to an even depth of 3,000 sequences. Four samples were discarded due to insufficient sequence coverage. For the second experiment, samples were rarified to an even depth of 45,000 sequences. One sample was discarded due to insufficient coverage. Alpha, and beta-diversity metrics were produced using QIIME [38]. Relationships between samples were visualized and evaluated using redundancy analysis (RDA) and principal coordinate analyses (PCoA) calculated from pairwise sample distances (weighted and unweighted UniFrac metrics) [42]. Significance tests were run using the compare_categories.py (ANOSIM, ADONIS, ANOVA, and RDA) and compare_distance_matrices.py (Mantel) scripts in QIIME [38]. To evaluate the most important abiotic factors in structuring the communities, a Best Subset of Environmental Variables with Maximum (Rank) Correlation with Community Dissimilarities (BEST) analysis was run in QIIME (*see vegan::bioenv*) [43].

## Results

Work for this study was accomplished in two experiments. First, we performed an experiment to identify variation in the microbial communities between roots and soil in three different *Cannabis* strains (Burmese, BooKoo Kush, and Sour Diesel), and second, an experiment designed to understand the nature and strength of plant cultivar-specificity between two different strains (White Widow and Maui Wowie) in two different soil types (with significant differences in edaphic variables). Triplicate samples were taken from each plant for both the rhizosphere and endorhiza, as well as for each of the two soil types.

### Both endorhiza and bulk soil microbiomes were significantly distinct from other sample types, and strain level differences were only observed in the endorhiza

**Figure 1:**
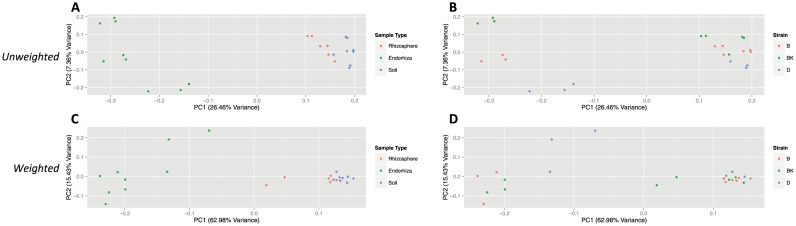
PCoA plots of microbial community similarity in first experiment for unweighted analysis (A–B) and weighted analysis (C–D). Plots for unweighted analysis are based on unweighted UniFrac distance, and demonstrate relationship between sample type (A), strain (B), and the major PC axes (PC 1 = 26.46% variance, PC 2 = 7.36% variance). Plots for weighted analysis are based on weighted UniFrac distances, and demonstrate relationship between sample type (C), strain (D), and the major PC axes (PC 1 = 62.98% variance, PC 2 = 15.43% variance). Abbreviations for strains are denoted by B (Burmese), BK (BooKoo Kush), and D (Sour Diesel).

In the first experiment, using unweighted UniFrac, beta-diversity comparisons of each individual sample type against all other sample types (**Fig. 1a**) yielded significant clustering of endorhiza (ADONIS: R^2^ = 0.26, p = 0.001) and bulk soil (ADONIS: R^2^ = 0.14, p = 0.001) samples from the other categories, but rhizosphere samples were not significantly different (ADONIS: R^2^ = 0.07, p = 0.07). Weighted UniFrac distances yielded similar results with endorhiza (ADONIS: R^2^ = 0.59, p = 0.001) and bulk soil (ADONIS: R^2^ = 0.29, p = 0.004) samples demonstrating significant differences from other sample types, but no significant differences for rhizosphere (ADONIS: R^2^ = 0.09, p = 0.10) samples. Division of all communities via strain (**Fig. 1b**) was not significant for weighted (ADONIS: R^2^ = 0.11, p = 0.25) or unweighted (ADONIS: R^2^ = 0.11, p = 0.15) analyses, however, division of endorhiza communities via strain was significant for both weighted (ADONIS: R^2^ = 0.59, p = 0.004) and unweighted (ADONIS: R^2^ = 0.39, p = 0.003) analyses. The abundance of *Methylophilus* explained a significant portion of this difference (FDR: p = 0.012), comprising 13% of the microbial community in the endorhiza of Bookoo Kush, 0.13% in Burmese and was absent in Diesel. Despite these significant differences, all endorhiza samples maintained a core community of *Pseudomonas, Cellvibrio, Oxalobacteraceae*, *Xanthomonadaceae*, *Actinomycetales*, and *Sphingobacteriales*. With the exception of the aerobic cellulytic bacterium *Cellvibrio,* all prevalent members of the core endorhiza community were well known endophytic bacteria [44], [45] primarily within the orders Gammaproteobacteria and Alphaproteobacteria, which supports observations from other plant systems [46], [47].

### Community composition across all samples was determined predominantly by soil properties, but differences in community structure (abundance) within endorhiza were driven by *Cannabis* cultivar

**Figure 2:**
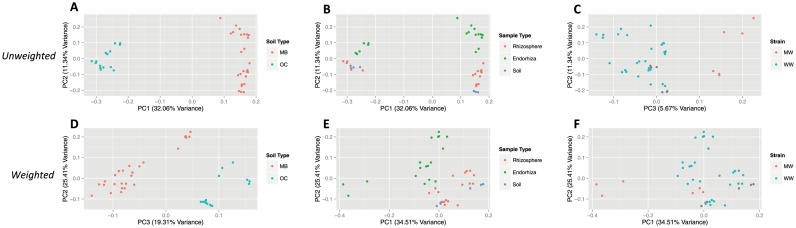
PCoA plots of microbial community similarity in second experiment for unweighted analysis (A–C) and weighted analysis (D–F). Plots for unweighted analysis are based on unweighted UniFrac distances, and demonstrate relationship between soil type (A), sample type (B), strain (C), and the major PC axes (PC 1 = 32.06% variance, PC 2 = 11.34% variance, PC 3 = 5.67% variance). Plots for weighted analysis are based on weighted UniFrac distances, and demonstrate relationship between soil type (D), sample type (E), strain (F), and the major PC axes (PC 1 = 34.51% variance, PC 2 = 25.41% variance, PC 3 = 19.31% variance). Note that PC 1 in the unweighted analysis is dominated by variation in soil type (A), but PC 1 in weighted analysis is dominated by strain (F). Grey points (Fig. 2c, 2f) represent bulk soil samples that aren't associated with either strain. Abbreviations for strains are denoted by MW (Mauie Wowie) and WW (White Widow), and abbreviations for soil type are denoted by MB (Mo-Bio soil) and OC (Orange County soil).

In the second experiment, using unweighted UniFrac, community beta diversity was significantly different between soil types (**Fig. 2a**) (ADONIS: R^2^ = 0.32, p = 0.001), among sample types (**Fig. 2b**) (ADONIS: R^2^ = 0.12, p = 0.005), and strains (**Fig. 2c**) (ADONIS: R^2^ = 0.10, p = 0.008). Cluster comparisons of each individual sample type against all other sample types (**Fig. 2b**) yielded significant differences for endorhiza (ADONIS: R^2^ = 0.10, p = 0.001) and rhizosphere (ADONIS: R^2^ = 0.05, p = 0.04) samples, but no significant differences for bulk soil (ADONIS: R^2^ = 0.04, p = 0.12) samples. Using weighted UniFrac, community beta diversity varied significantly by soil type (**Fig. 2d**) (ADONIS: R^2^ = 0.21, p = 0.001), sample type (**Fig. 2e**) (ADONIS: R^2^ = 0.27, p = 0.001), and strain (**Fig. 2f**) (ADONIS: R^2^ = 0.27, p = 0.001). Cluster comparisons of each individual sample type against all other sample types (**Fig. 2e**) yielded significant differences for endorhiza (ADONIS: R^2^ = 0.26, p = 0.001) and rhizosphere (ADONIS: R^2^ = 0.13, p = 0.001) samples, with mixed results for bulk soil samples (ADONIS: R^2^ = 0.06, p = 0.054; ANOSIM: −0.012, p = 0.459; RDA: F = 2.41, p = 0.045).

**Figure 3:**
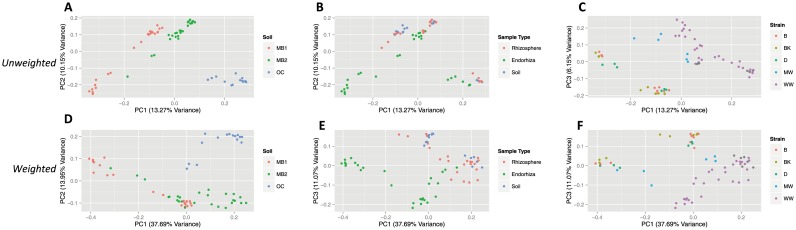
PCoA plots of microbial community similarity in pooled experiments for unweighted analysis (A–C) and weighted analysis (D–F). Plots for unweighted analysis are based on unweighted UniFrac distance, and demonstrate relationship between soil type (A), sample type (B), strain (C), and the major PC axes (PC 1 = 13.27% variance, PC 2 = 10.15% variance, PC 3 = 6.15% variance). Plots for weighted analysis are based on weighted UniFrac distances, and demonstrate relationship between soil type (D) sample type (E), strain (F), and the major PC axes (PC 1 = 37.69% variance, PC 2 = 13.95% variance, PC 3 = 11.07% variance). Abbreviations for strains are denoted by B (Burmese), BK (BooKoo Kush), D (Sour Diesel), MW (Mauie Wowie) and WW (White Widow). Abbreviations for soil type are denoted by MB1 (Mo-Bio soil from the first experiment), MB2 (Mo-Bio soil from the second experiment) and OC (Orange County soil).

Pooling the first and second experiments together, division of all communities via soil type (**Fig. 3a**) (ADONIS: R^2^ = 0.196, p = 0.001), sample type (**Fig. 3b**) (ADONIS: R^2^ = 0.086, p = 0.001), and strain (**Fig. 3c**) (ADONIS: R^2^ = 0.178, p = 0.001) were highly significant for all tests using unweighted UniFrac. Cluster comparisons of each individual sample type against all other sample types yielded significant results for **endorhiza** samples (ADONIS: R^2^ = 0.069, p = 0.001), and mixed results for rhizosphere (ADONIS: R^2^ = 0.034, p = 0.004; ANOSIM: R = 0.005, p = 0.365; RDA: F = 2.17, p = 0.001) and bulk soil samples (ADONIS: R^2^ = 0.031, p = 0.005; ANOSIM: R = −0.032, p = 0.628; RDA: F = 2.00, p = 0.003). Likewise, using weighted UniFrac, the division of all communities via soil type (**Fig. 3d**) (ADONIS: R^2^ = 0.323, p = 0.001), sample type (**Fig. 3e**) (ADONIS: R^2^ = 0.229, p = 0.001), and strain (**Fig. 3f**) (ADONIS: R^2^ = 0.301, p = 0.001) was highly significant for all tests. Cluster comparisons of each individual sample type against all other sample types yielded significant results for **endorhiza** samples (ADONIS: R^2^ = 0.215, p = 0.001), and mixed results for rhizosphere (ADONIS: R^2^ = 0.093, p = 0.002; ANOSIM: R = 0.045, p = 0.129; RDA: F = 6.36, p = 0.002) and bulk soil samples (ADONIS: R^2^ = 0.057, p = 0.008; ANOSIM: R = −0.041, p = 0.691; RDA: F = 3.76, p = 0.006).

### Soil followed by strain had the largest affect on OTU abundances, but strain showed no impact on OTU presence/absence

For individual OTUs, both unweighted (g-test) and weighted (ANOVA) analyses showed that soil type had the strongest influence over significant OTU differences (**Table 2**). While strain showed a larger effect than sample type for weighted OTU differences, there were no significant unweighted OTU differences between strains, further suggesting the importance of strain in structuring OTU abundances - rather than OTU presence/absence.

**Table 2:** Number of significant OTUs for soil type, sample type, and strain.

	Weighted	Unweighted
**Soil Type**	690	657
**Sample Type**	51	11
**Strain**	71	0

Results of both unweighted (g-test) and weighted (ANOVA) analyses using FDR multiple test correction.

**Figure 4:**
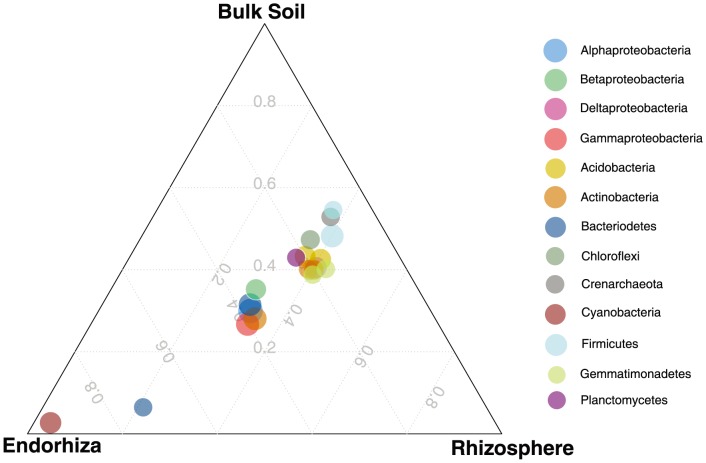
Ternary plot of distribution of bacterial taxonomic groups among sample types in the second experiment. Size of circles proportional to the log of the total abundance, taxonomic groups are all phylum-level, except for Proteobacteria, which is by class.

As suggested by the two-tier model [33], our results demonstrate a decrease in abundance of *Acidobacteria* and an increase of *Proteobacteria* and *Actinobacteria* relative to the rhizosphere and bulk soil (**Fig. 4**). Furthermore, the most significant OTU abundance difference between sample types was the decrease in *Acidobacteria* from the order iii1-15 in endorhiza samples (Bonferroni-corrected ANOVA: p = 1.12e-7). Of the 51 OTUs significantly differentiating between sample types, the 17 OTUs which increased in abundance within the *Cannabis* endorhiza relative to rhizosphere were predominantly *Proteobacteria*, including several from the *Rhizobiales* order. Mean abundance of the 51 OTUs were highly correlated between bulk soil and rhizosphere samples (Pearson's rho: 0.92), versus a lower correlation between rhizosphere and *Cannabis* endorhiza (Pearson's rho: 0.63), and even lower between bulk soil and *Cannabis* endorhiza (Pearson's rho: 0.42).

Significant OTU abundance differences between strains were composed mostly of differences in *Proteobacteria*, notably *Pseudomonadales*, *Burkholderiales*, *Sphingomonadales*, and *Rhizobiales*. Apart from *Proteobacteria*, *Bacteroidetes* orders *Sphingobacteriales* and *Flavobacteriales* were also responsible for several significant OTU differences between *Cannabis* strains. Intriguingly, one of the significant OTUs between strains was the prevalence of *Sphingomonas wittichii* in the Maui Wowie strain, which in some contexts can metabolize phenazine-1-carboxylic acid and has been implicated in increased survival in soil environments.

### Bulk soil and rhizosphere microbiomes are more similar to each other than to endorhiza microbiomes

**Figure 5:**
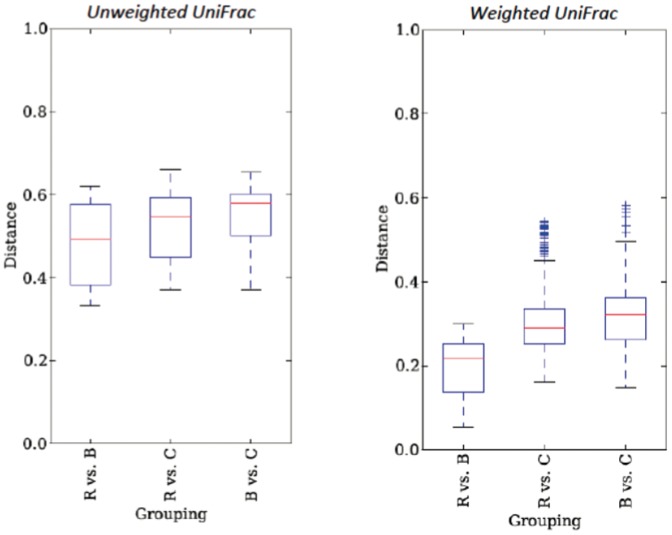
Box plots of beta-diversity distances between communities for both weighted and unweighted analyses. Initials (i.e. B vs. C) stand for comparisons of beta-distances for samples within groups (R =  rhizosphere, C =  *Cannabis* endorhiza, B =  bulk soil).

(Fig. 5)Beta distances between rhizosphere and bulk soil communities were significantly lower than distances between rhizosphere and endorhiza communities for both unweighted and weighted analyses (*unweighted*: t = −4.59, p<0.001 *weighted*: t = −11.82, p<0.001). Beta distances between rhizosphere and bulk soil communities were significantly lower than distances between bulk soil and endorhiza communities for both unweighted and weighted analyses (*unweighted*: t = −5.15, p<0.001; *weighted*: t = −11.56, p<0.001). Beta distances between rhizosphere and endorhiza communities were not significantly different from distances between bulk soil and endorhiza communities for both unweighted and weighted analyses (*unweighted*: t = −2.10, p = 0.109; *weighted*: t = −2.23, p = 0.078).

### Endorhiza share more OTUs with the soil they are grown in than with another soil in which the same strain is grown

Previously, a two-step process of root colonization, first fueled by rhizodeposition and followed by fine-tuning by host genotype has been posited [34], [35], [48]. This two-step selection model was tested by pooling samples by *Cannabis* strain and analyzing the core microbiome within each strain. As bulk soils are the putative source of microbes for the plant, endorhiza communities would be expected to share more OTUs with their own soil than with another. White Widow was grown in two different soils, and roots shared more OTUs with the soil they were grown in than with the different soil in which the different white widow plant was grown. The number of shared OTUs between endorhiza and their own soil (n = 45, mean  = 2934) was significantly greater (t = −10.05, p = 1.209e-15) than the number of shared OTUs between endorhiza and the other soil (n = 45, mean  = 2162).

### Cannabinoid concentration and composition was significantly correlated to structure of endorhiza communities

Each plant in the second experiment was tested for a variety of cannabinoids, including delta-9-tetrahydrocannibinol. Cannabinoid data associated with the plants was used in Mantel tests to understand the potential biochemical associations with community composition or structure; with significant differences between strains (unweighted; *r-stat*: 0.863, *p-value*  = 0.001). However, due to higher THC composition and concentration in plants from one of the soil types, THC variables were also significantly correlated to the soil edaphic variables, and as such any association between microbiota and THC is very hard to disassociate from soil physicochemical variables.

### Edaphic factors were strongly linked to structure of microbial communities in rhizosphere and endorhiza communities

In both experiments, edaphic data associated with the plants was used in Mantel tests to understand the effect of edaphic factors on structuring bulk soil, rhizosphere, and *Cannabis* endorhiza communities. For all experiments, the soil texture was defined as a sandy loam, with significant differences in clay and other edaphic factors (**Table 2**) between the two soil types in the second experiment, and the soil types used in the first experiment. For both weighted and unweighted UniFrac distances (all samples pooled in the analysis), all edaphic factors tested were significantly correlated with community beta-diversity (p = 0.001). For the weighted analysis, Nitrogen had the strongest effect in structuring the communities (*r-stat*: 0.465, *p-value*  = 0.001), followed by salinity (*r-stat*: 0.437, *p-value*  = 0.001), Carbon (*r-stat*: 0.330, *p-value*  = 0.001), water content (*r-stat*: 0.281, *p-value*  = 0.001), and pH (*r-stat*: 0.221, *p-value*  = 0.001). For the unweighted analysis, the relative importance of the edaphic factors remained the same, with Nitrogen as the most important (*r-stat*: 0.630, *p-value*  = 0.001), followed by salinity (*r-stat*: 0.620, *p-value*  = 0.001), Carbon (*r-stat*: 0.512, *p-value*  = 0.001), water content (*r-stat*: 0.466, *p-value*  = 0.001), and pH (*r-stat*: 0.292, *p-value*  = 0.001). Running a BEST analysis, the variance in community data is optimally explained by three edaphic factors; Nitrogen, Carbon, & Water (rho = 0.632).

### Alpha diversity peaks in bulk soil and declines with the transitions into the rhizosphere and endorhiza microbiomes

**Figure 6:**
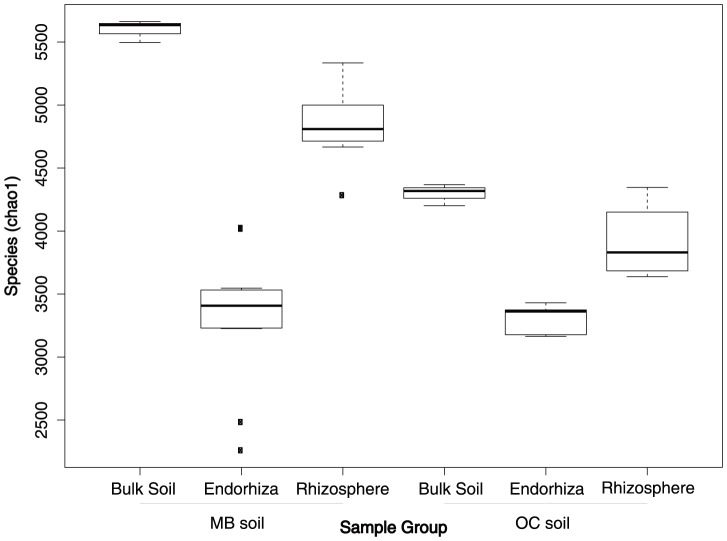
Box plots of alpha diversity (observed species) for endorhiza, rhizosphere, and bulk soil from two separate soil types in the second eperiment. MB  =  Mo-Bio soil, OC  =  Orange County soil. Note the significant differences between alpha diversity in the bulk soil and rhizosphere but negligible differences between endorhiza alpha diversity between soil types.

(Fig. 6) Observed species and chao1 alpha-diversity metrics from the second experiment demonstrated a slight reduction in alpha diversity from bulk soil (*chao1*: µ = 4947; σ = 717) to rhizosphere samples (*chao1*: µ = 4525; σ = 542), followed by a dramatic reduction in alpha diversity from rhizosphere to endorhiza (*chao1*: µ = 3321; σ = 420). Although diversity was significantly higher in MB bulk soil (*chao1*: µ = 5597; σ = 89) and rhizosphere (*chao1*: µ = 4859; σ = 286) in comparison to OC bulk soil (*chao1*: µ = 4296; σ = 85) and rhizosphere (*chao1*: µ = 3913; σ = 290), diversity of MB endophytes (*chao1*: µ = 3325; σ = 517) was not significantly different from that of the OC endosphere (*chao1*: µ = 3311; σ = 112). Despite much shallower sequencing in the first experiment, the same pattern was recovered, with both species richness and chao1 diversity index highest in bulk soil (*chao1*: µ = 2010.7, σ = 146.2), slightly lower in the rhizosphere (*chao1*: µ = 1837.2, σ = 114.0), and lowest in the endorhiza (*chao1*: µ = 916.1, σ = 161.7).

Curiously, when samples from both experiments were pooled and rarified to the level of the first experiment, alpha diversity of the endosphere from the first experiment was greatly reduced (*chao1*: µ = 916.1, σ = 161.7) in comparison to the endospheres grown in MB soil (*chao1*: µ = 1413, σ = 280.1) and OC soil (*chao1*: µ = 1374, σ = 64.4). Although this might be explained by decreased diversity in the MB soil from the first experiment (MB1), comparisons of diversity between rarified samples demonstrate MB1 bulk soil had intermediate diversity (*chao1*: µ = 2010.7, σ = 146.2) relative to the MB bulk soil (*chao1*: µ = 2319.1, σ = 124.3) and the OC bulk soil (*chao1*: µ = 2004.8, σ = 118.6). This reduction in alpha diversity in the endosphere from the first experiment is consistent with the early stages of root decay following the harvesting of the plant.

### Composition of endorhiza communities in the first experiment suggest potential root decay

After analysis of the data from the first experiment yielded high abundances (greater than 10% of taxonomy assigned reads) of the known cellulytic bacterium *Cellvibrio*, we sought to investigate the possibility that *Cellvibrio* was an indication of root decay rather than its unexpected presence as a member of the endophytic core community. This was of particular interest because the samples were taken 8 weeks post-harvest. Comparisons of relative abundances of *Cellvibrio* between the first and second experiments yielded rather convincing results demonstrating the early stages of root decay despite significant cultivar-specificity within the samples. Specifically, the relative abundance of *Cellvibrio* within the endosphere of the first experiment was 16.9% (σ = 13.0%, N = 9) versus 0.095% (σ = 2.7%, N = 18) in the endosphere of the second experiment.

## Discussion

Recent literature has suggested a two-step selection model for the endorhiza, where bulk-soil microbial communities are filtered by increased concentration of rhizodeposits, followed by convergent host genotype-dependent selection on endophytic communities [34], [35], [48]. Results from both experiments support many of the expectations produced by this model. Most importantly, the principal coordinate analysis (PCoA) plots for the second experiment demonstrate highly significant clustering patterns. First, soil type is the main determinant of PC1 (32.06%) for the unweighted analysis of the second experiment, revealing that soil is undoubtedly the most important factor in all samples for determining what microbes are present. Second, communities within both soil types demonstrate a similar community shift from bulk soil to endorhiza samples along PC2 (11.34%), which is dominated by differentiation between sample types. Specifically, endorhiza samples have high, positive values along PC2, rhizosphere samples have intermediate values, and bulk soil samples have more negative values. Third, *Cannabis* strain is the main determinant of PC1 (34.51%) for the weighted analysis of all samples in the second experiment, suggesting that convergent host genotype-dependent selection acts through controlling community structure (abundance) more than composition. PCoA results exhibit how all sample types form significantly differentiated clusters in weighted analyses but that only rhizosphere and endorhiza samples form significantly differentiated clusters in unweighted analyses, suggesting niche-filtering of microbes in rhizosphere and endorhiza samples from bulk soil. Furthermore, there were no significant segregating OTUs based on unweighted analysis between cultivars in endorhiza and rhizosphere samples in the second experiment, however there were 71 when abundance was accounted for. This differs greatly from the 657 OTUs that significantly differ between soil types in the same dataset. Testing of the two-step selection model with pairwise comparisons of shared OTUs between endorhiza and bulk soil samples also validated the hypothesis that a portion of the endophytic microbes are inherited and selected from the surrounding soil, showing significantly more OTU overlap between endorhiza and their own bulk soil compared to endorhiza and foreign bulk soil.

Given the results from the second experiment strongly suggesting that *Cannabis* cultivars have important structuring effects on *both* rhizosphere and endorhiza samples, it may seem troubling that results from the first experiment do not suggest this for the rhizosphere samples. However, differences in *Cellvibrio* abundance between experiments show that root decay could have diminished the rhizosphere effect, thus diminishing this potential signal. Sampling for the first experiment was done post-harvest, when plant tissues were undergoing senescence and decay, while samples for the second experiment were taken from actively growing plants. Considering the extensive work demonstrating the importance of plant growth stage on the microbiota [21], [49], as well as the plant-soil feedbacks identified in structuring belowground microbial communities [50], [51], the differences between the first and second experiments are unsurprising. The similarities, however, are surprising. In particular, that cultivar-specificity could be identified in the microbiota within the endorhiza samples in the first experiment without any input of cultivar-specific metabolites from the living plant for weeks.

Although we have presented several highly significant findings supporting expectations of the two-step selection model, some expectations remain to be validated. Specifically, although the mean beta-diversity distances indicate that rhizosphere and endorhiza samples are closer than bulk soil and endorhiza samples, this difference was not significant and thus provides little evidence for the first differentiation step of the two-step selection model [34], [35], [48].

Future work with the *Cannabis* microbiome should focus on elucidating the role of cultivar on rhizosphere, as well as what aspects of host genotype are producing the structure observed across *Cannabis* strains. Increased testing of cannabinoids and decoupling this variation from edaphic factors will improve our understanding of the importance of cannabinoid production in structuring endorhiza communities. Sampling a time series of endorhiza communities across several plants may help us to understand natural variation in the endorhiza during the reproductive cycles of *Cannabis*. Understanding this natural variation will help direct future mechanistic studies aimed at using microbial communities to increase plant fitness, suppress disease, or augment desired metabolite production.

## Supporting Information

Table S1 **THC-testing data.**
(DOCX)
