# Correction: Understanding Cultivar-Specificity and Soil Determinants of the *Cannabis* Microbiome

**DOI:** 10.1371/journal.pone.0107415

**Published:** 2014-09-02

**Authors:** The PLOS ONE Staff

The seventh author’s name and affiliation are incorrect. The correct name is: Joshua A. Hartsel. The correct affiliation is: Delta-9-Technologies, 2534 State Street Offices Suite 458, San Diego, CA 92101.

## References

[1] Winston ME, Hampton-Marcell J, Zarraonaindia I, Owens SM, Moreau CS, et al (2014) Understanding Cultivar-Specificity and Soil Determinants of the Cannabis Microbiome. PLoS ONE 9(6): e99641 doi:10.1371/journal.pone.0099641 24932479 10.1371/journal.pone.0099641 PMC4059704

