# Supplementary material for: Understanding Cultivar-Specificity and Soil Determinants of the Cannabis Microbiome
Source: PLoS One. 2014 Jun 16;9(6):e99641. doi: 10.1371/journal.pone.0099641 (PMC4059704; doi:10.1371/journal.pone.0099641)
Supplement: Table S1 — THC-testing data. (DOCX) [file pone.0099641.s001.docx]

**Supplementary Information**

**Table S1: THC-testing data**

|  |  | **% of dry sample** | | | |  |
| --- | --- | --- | --- | --- | --- | --- |
| **Strain** | **ID** | **THC** | **THCA** | **CBD** | **CBN** | **Total Cannabinoids** |
| White Widow | WW.OC.1 | 0.38 | 21.1 | 0.05 | 0 | 22.29 |
| White Widow | WW.OC.2 | 0.49 | 20.01 | 0.03 | 0.09 | 21.08 |
| White Widow | WW.MB | 0 | 3.48 | 0 | 0.01 | 3.53 |
| Mauie Wowie | MW.MB | 0 | 3.01 | 0 | 0 | 3.04 |
